# Elephant Sound Classification Using Deep Learning Optimization

**DOI:** 10.3390/s25020352

**Published:** 2025-01-09

**Authors:** Hiruni Dewmini, Dulani Meedeniya, Charith Perera

**Affiliations:** 1 Department of Computer Science and Engineering, University of Moratuwa, Moratuwa 10400, Sri Lanka; h.u.dewmi@gmail.com (H.D.); dulanim@cse.mrt.ac.lk (D.M.); 2 School of Computer Science and Informatics, Cardiff University, Cardiff CF24 3AA, UK

**Keywords:** artificial intelligence, audio processing, deep learning, elephant vocalization, optimization, resource constrained

## Abstract

Elephant sound identification is crucial in wildlife conservation and ecological research. The identification of elephant vocalizations provides insights into the behavior, social dynamics, and emotional expressions, leading to elephant conservation. This study addresses elephant sound classification utilizing raw audio processing. Our focus lies on exploring lightweight models suitable for deployment on resource-costrained edge devices, including MobileNet, YAMNET, and RawNet, alongside introducing a novel model termed ElephantCallerNet. Notably, our investigation reveals that the proposed ElephantCallerNet achieves an impressive accuracy of 89% in classifying raw audio directly without converting it to spectrograms. Leveraging Bayesian optimization techniques, we fine-tuned crucial parameters such as learning rate, dropout, and kernel size, thereby enhancing the model’s performance. Moreover, we scrutinized the efficacy of spectrogram-based training, a prevalent approach in animal sound classification. Through comparative analysis, the raw audio processing outperforms spectrogram-based methods. In contrast to other models in the literature that primarily focus on a single caller type or binary classification that identifies whether a sound is an elephant voice or not, our solution is designed to classify three distinct caller-types namely roar, rumble, and trumpet.

## 1. Introduction

Elephant sound classification plays a crucial role in forest observatory research, particularly in understanding elephant behavior, communication efforts, and their ecological impact [1,2]. Elephant vocalizations indicate the presence of specific species within a forest ecosystem. By classifying these sounds, researchers can track elephant populations and their movements, which is essential for understanding biodiversity dynamics in forest habitats. Elephants utilize a complex vocalization that includes low-frequency rumbles and other sounds to communicate over long distances [3]. Different caller types such as chirps, roars, trumpets, and rumbles reflect various behaviors such as emotional states, mating calls or alarm signals [4]. Analyzing these vocalizations helps in understanding social structures and interactions within elephant herds. In addition, the real-time elephant behavior monitoring by distinguishing sounds associated with conflict or group defense can help prevent human–elephant conflicts and poaching incidents. Thus, monitoring the changes in vocal patterns through sound classification can inform conservation strategies, habitat management, and restoration practices [2,5].

Recent advancements in deep learning techniques have improved the performance of environment sound classification focusing on the health of forest ecosystems [6]. For instance, using Convolutional Neural Networks (CNNs) allows for the effective extraction of relevant features from elephant vocalizations, enhancing the reliability of monitoring systems [7]. The development of comprehensive datasets [8], and optimized techniques to deploy the solutions in resource-constrained environments [9,10,11], are critical for developing robust monitoring tools that can be deployed in real-world scenarios.

Elephant vocalizations have been investigated for their functional significance and production mechanisms, as the basis for developing automated acoustic detection methods. Most of the traditional approaches target the analysis of specific call types, which were pre-segmented manually. Generally, the classification algorithms primarily rely on extracted sound features, that consume computational complexity. Some of the feature extraction techniques include Mel-frequency Cepstral Coefficients (MFCC) [7,12,13,14], Spectral Subband Centroids (SSC), Linear Predictive Coding (LPC), and Filter Bank Energies (FBE) [15]. However, deploying such models onto lightweight edge devices often poses challenges due to the additional time and computational resources required for preprocessing. Hence, our objective is to explore the feasibility of training machine learning models directly on raw audio data, aiming to ascertain whether comparable accuracy levels can be achieved without the need for traditional feature extraction methods.

The synergy between edge computing and AI significantly enhances elephant sound classification efforts in forest observatory. By enabling monitoring, improving data efficiency, and offering scalable solutions, this technology is pivotal in advancing conservation strategies aimed at protecting elephants from threats like poaching, habitat loss, and observing behaviors [16,17]. This approach leverages real-time data processing at the source locally [9], which is essential for effective conservation strategies with quick decision-making, providing insights into the health and well-being of elephants. Modern AI solutions encompass various models to process raw audio data directly without a feature extraction process [11], and classify sounds with model optimization techniques [9,10]. Thus, with the advancement of bioacoustics processing, leveraging raw audio data directly could lead to effective species identification in wildlife monitoring. This approach eliminates the need for converting audio into spectrogram-like representations or handcrafted features, allowing algorithms to autonomously identify pertinent components within the audio waveform [18]. However, the process of selecting appropriate features for analysis is intricate and lacks a universally optimal solution, due to the inherent complexity of raw audio data [19].

In the domain of elephant vocalization analysis, there has been limited exploration into the direct processing of raw audio data. Predominantly, various feature extraction techniques have been employed before training machine learning algorithms. In our investigation, we aim to bypass preprocessing stages and directly input raw audio data into machine learning models to assess the feasibility and efficacy of training on unprocessed audio signals. Our research study presents a novel approach centered on raw audio processing in the domain of elephant sound classification focusing on reducing the computational complexity and improving scalability; thereby paving the way for the development of more efficient and effective solutions in this domain. The main contributions of this paper are as follows:

- Explore deep learning techniques to process elephant caller types.
- Assess the impact of augmentation levels in elephant sound classification.
- Design and develop a lightweight solution optimized for elephant sound identification.
- Conduct a comparative analysis of direct raw audio classification and spectrogram-based elephant sound classification.
- Evaluate the impact of raw audio processing, demonstrating that it can achieve accuracy and inference times comparable to existing methods.

The rest of the paper is organized as follows: the related work is discussed in Section 2. The proposed model design and methodology are detailed in Section 3, and the results of the evaluations are presented in Section 4. The study contributions, comparison with the existing studies, limitations, and future extensions are discussed in Section 5. Finally, Section 6 concludes the paper.

## 2. Related Work

Elephant vocalizations are complex and context-dependent, reflecting emotional states and social dynamics. Understanding these calls enhances the analysis of elephant communication and behavior in their natural habitats. The classification of elephant vocalizations encompassed an expansive spectrum of call types that spanned over distinct categories, including growl, squeak, long roar-rumble, long roar, rumble, bark, trumpet, roar-rumble, roar, squeal, croak rumble, chirp-rumble, and musth cry-rumble [20]. Among them, rumbles are more common and heard during relaxed social interactions and long-distance communication. Chirps are mainly associated with Asian elephants and are used during supportive communications and group assembling. Trumpeting is often associated with excitement, distress, or aggression. Similarly, roaring is typically used during high distress or aggression situations and also occurs during joyful reunions, indicating a complex emotional range [3,21]. Thus, the identification of different elephant caller types helps to understand animal behaviour and the decision-making process in forest observatory.

In the literature on sound classification, feature extraction plays a significant role by enabling the derivation of key audio characteristics such as short-time energy, bandwidth, and zero-crossing rate. This process effectively reduces the dimensionality of the audio input vector while preserving essential discriminative features. The feature extraction process for elephant sounds differs primarily in terms of the frequency range, temporal patterns, complexity, environmental considerations, and biological context. Different feature extraction techniques have been used to transform audio data into visual representations, which are fed to machine learning models for classification. Among several techniques such as Gammatone Frequency Cepstral Coefficient, Mel-frequency cepstral coefficients (MFCC), linear prediction cepstral coefficients (LPCC), or Linear Predictive Coding (LPC) [4,7], and MFCC has emerged as a widely used feature extraction technique [22]. For instance, Leonid et al. [7], applied data preprocessing on the Elephant Voice Dataset using Min-Max scalar and standard scalar feature extraction techniques. Moreover, robust models were proposed to accurately classify forest observatory sound data by combining several feature extraction techniques [10]. The choice of the feature extraction technique depended on the particular specifications of the sound analysis undertaking and their characteristics [23].

In practice, machine learning algorithms such as Neural Networks, Support Vector Machine (SVM) [24], Hidden Markov Models [4], and Gaussian Mixture Models were commonly used in species recognition tasks [25]. With the development of deep learning and machine learning models, CNN algorithms and ensemble approaches have garnered greater popularity [26], despite slightly lower accuracy rates reported in the sound classification domain. Table 1 gives a summary of related studies. Although most of the literature focuses on binary classification tasks by Geldenhuys et al. [27], it delved into identifying different caller types. They performed a comparative study to assess several models including Logistic Regression, SVM, XGBoost, MLP, AlexNet, and ResNet, and the Audio Spectrogram Transformer (AST) in one-to-one sequence configuration over Asian and African elephant vocalization. They utilized dimensionality reduction and feature extraction techniques, such as MFCC and Mel-spectral configurations, to optimize performance. Their work addressed both binary classification for frame-wise call detection and multi-class classification for five vocalization types. The highest performance is shown by the transformer-based AST model, with a precision of 73% for the Asian elephant dataset from Sri Lanka. This model handles complex multi-class and sub-call classifications and achieves state-of-the-art performance.

Advancements in deep learning, such as CNN-LSTM architectures, improved temporal modeling, yielding better recall rates and precision. The introduction of spectrogram-based methods, such as STFT and Mel-spectrograms, marked a significant shift toward the use of richer audio features, as seen in models like YAMNet [28]. In another study by Silva et al. (2023) [29], they utilized the publicly available Asian Elephant Vocalization Dataset, focusing exclusively on rumbles. They applied wavelet-based signal decomposition and reconstruction techniques before extracting features from combinations such as chroma CQT, chroma CENS, Mel spectrogram, MFCC, and spectral contrast. Using an SVM classifier, they achieved a precision and recall of 84% for elephant rumble detection.

Moreover, Leonid et al. [7] proposed a parallel CNN model to classify African elephant sounds. The approach utilizes vocal feature sets, including MFCC, LPC, and FBE, passed through multi-input layers connected to parallel convolution layers. This study showed a high accuracy of 96.2%, with a computation time of 11.89 s. Similarly, Bjorck et al. [25], proposed a solution that combines automatic detection of African elephant vocalizations with data compression to enable efficient, large-scale acoustic monitoring. They used spectrograms to visually represent sound frequencies over time, focusing on low-frequency calls, to differentiate elephant calls from background noise. They have integrated audio compression algorithms with their CNN-LSTM to reduce the model size without lowering the accuracy.

Audio signals were represented in the time domain as waveforms, depicting changes in amplitude over time. The direct processing of raw audio, bypassing the need for preprocessing or feature extraction, using neural networks have garnered significant attention in forest observatory sounds classification [9,11]. Subsequently, researchers aimed to unlock new insights and applications in raw audio processing in resource-constrained edge devices, paving the way for efficient and accurate sound classification systems.

In another point of view, several deep learning solutions have been developed to address environmental sound classification by processing raw audio directly [9,11,30]. Among them, YAMNet, Yet Another Mobile Network, is a neural network for sound classification, that utilizes the MobileNetV1 architecture, which is optimized for performance and efficiency, making it suitable for various applications in environmental sound recognition [30]. It takes raw audio data as input, making it versatile and suitable for classification process. ACDNet, Deep Acoustic Networks on Extremely Resource-Constrained Devices, is another model [9], for raw sound classification. ACDNet relies on convolutional layers to directly extract features from raw audio signals, bypassing the conventional reliance on spectrogram-based preprocessing. This streamlined approach simplifies the model architecture by facilitating efficient utilization of computational resources, making it particularly well-suited for deployment on resource-constrained devices. ESC-NAS, Environment Sound Classification with Neural Architecture Search [11], is another hardware-aware NAS approach. They have proposed a cell-based search space to design and develop deep convolutional neural network architectures, specifically tailored to handling raw audio inputs for environmental sound classification applications under limited computational resources.

Accordingly, training deep learning models requires substantial computational power and memory, which may not always be accessible in field settings. This limitation can hinder real-time processing capabilities that are crucial for immediate conservation decision-making. This study mainly considers the direct processing of raw elephant sound data, without any intermediate preprocessing steps. This approach was tailored to cater to the necessities of real-time elephant caller-type identification applications. Notably, the existing landscape of research lacks models specifically trained for identifying elephant voices from raw audio data. Hence, our investigation delves into four models capable of raw audio processing. These models were meticulously customized to accommodate our training datasets, without relying on pre-trained weights and pre-processing steps.

## 3. System Design and Methodology

**Figure 1:**
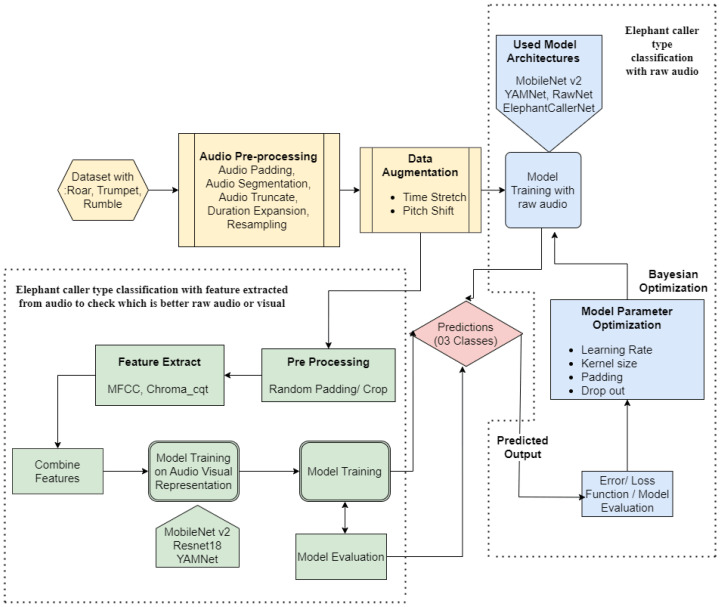
Working Flow of Research Implementation.

The proposed elephant sound classification pipeline consists of two main modules as shown in Figure 1. First, the elephant caller type classification with raw audio, which is the main emphasis of this study. Secondly, the elephant caller type classification with feature extraction utilizing spectrogram processing for comparison purposes of the proposed model, as shown in Figure 1. This section describes the workflow with the scientific contribution in detail.

### 3.1. Data Materials

In general, there is a limited availability of publicly accessible elephant vocalization datasets that encompass all call types. Analytically, extremely low-frequency call types pose significant challenges in analysis. Therefore, we focused on rumble, trumpet, and roar caller types in this study. The selected caller types covering a wide range of behaviors are described as follows [3,4,21]:

- Rumble: Elephant communication often relies on low-frequency vibrations called “rumbles”, produced through vocal cord vibration and altered by resonance within an elephant’s head or trunk structure. Rumbles play multiple functions such as maintaining social cohesion among group movements as well as conveying emotions like excitement distress or arousal.
- Trumpet: This is a trumpet-like calls produced by forcing air through an elephant’s trunk to produce loud, resonant noises that resonate loudly and frequently. Elephants use trumpets for various reasons such as alarm calls to warn potential threats or show dominance during social interactions and long-distance communication; their distinct nature adds much excitement for researchers and wildlife enthusiasts.
- Roar: Elephant roars are low-frequency vocalizations made during intense social interactions such as mating rituals or hostile encounters that serve to intimidate rivals, establish dominance, or attract potential mates.

Elephant vocalizations exhibit significant variability across different regions, genders, and ages. In this study, we focus on elephants from the Asian region and limit our dataset to adult elephants. We collected 235 elephant sound recordings from various repositories to create the dataset, including the Asian Elephant Vocalizations Dataset [31], and ElephantVoices Dataset [32]. Main portion of the elephant dataset was extracted from the Asian Elephant Vocalizations Dataset which contains 57.5 h of audio recordings collected over 18 months at Uda Walawe National Park in Sri Lanka, featuring 14 distinct call types. The ElephantVoices Dataset encompasses 23 call-type contexts associated with specific elephant behaviors and constellations. Other data sources include YouTube, and SoundCloud [33].

Subsequently, we assembled a dataset comprising 77, 63, and 95 audio clips for the caller types of roar, trumpet, and rumble, respectively. We extracted 6 s duration audio clips to ensure uniformity, eliminating silent intervals and extraneous audio segments devoid of elephant vocalizations. This duration is sufficient to capture significant portions of the vocalizations across all categories, retaining essential acoustic features that characterize different caller types. Additionally, a 6 s duration balances computational efficiency with sufficient content representation, as processing longer segments would require more resources and time. Techniques such as audio padding, truncation, time stretching, and audio repetition are undertaken for a balanced distribution of data. By padding shorter samples, we ensure no loss of original audio information, while trimming longer samples to 6 s retains the most relevant segment, maximizing feature representation.

Subsequently, the dataset was stratified into distinct classes, comprising train, validation, and test sets, distributed in a ratio of 80:10:10, respectively, before augmentation. Table 2 provides an overview of the dataset post-cleaning and preprocessing stages, delineating the number of audio files retained after pre-processing.

### 3.2. Data Augmentation

Augmentation techniques including time stretching and pitch shifting were then applied to the audio files, ensuring balanced representation across all classes and expanding the dataset to gain exposure to additional variations that help generalization and performance on unseen data sets.

Subsequently, two augmentation scenarios are utilized to assess the impact of augmentation on model performance. First, a minimum set of data augmentations is applied to the training set to generate a moderately expanded dataset for class balancing. For the second approach we applied several augmentation methods to significantly increase the dataset’s size and diversity. Here, our secondary objective was to compare the model performance that is trained on datasets with different levels of augmentation. It is important to note that excessive augmentation can adversely affect the quality and integrity of the dataset. Therefore, our focus was to evaluate how augmentation validates the audio processing, ensuring that it enhances the dataset without compromising its reliability.

Table 3 summarizes data distribution among train, validation, and test sets after data augmentation with both approaches. Here, the first approach focuses on class balancing, applying time stretch and pitch shift. The second approach focuses on increasing the dataset. Here, each audio file in the training and validation datasets was augmented four times using time stretching, pitch shifting, a combination of both methods and Gaussian noise.

### 3.3. Solution Design

**Figure 2:**
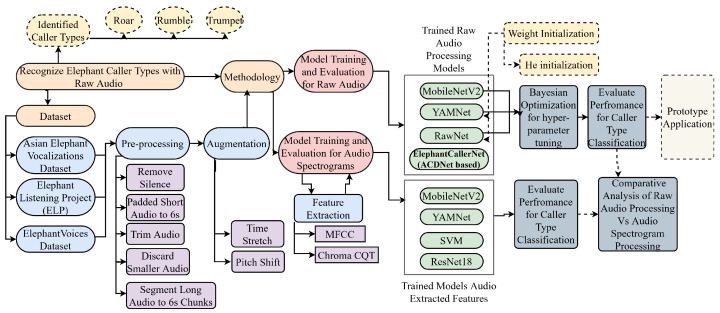
System Workflow.

As shown in Figure 2, we present a comprehensive methodology for recognizing different types of elephant calls, specifically focusing on roar, rumble, and trumpet caller types. The datasets, pre-processing and augmentation modules were described in the previous section. For the main methodology, we followed two pipelines, where (1) the first approach performs the classification on raw audio and (2) second the approach trains the models on the spectrograms obtained after the feature extraction process.

The first approach, raw audio processing involved models like MobileNetV2, YAMNet, RawNet, and the proposed ACDNet-based model named ElephantCallerNet. Notably, the existing models have not specifically designed for raw elephant audio data in the literature. Only MobileNetV2 utilized pre-trained weights; all other models were trained from scratch on the audio data. Consequently, we experimented with different hyperparameter tunings and weight initializations to achieve optimal accuracy for some models. For instance, *He Initialization* is designed for layers that use ReLU activations. It ensures the consistency of activation variances is maintained stable across layers, which is crucial for training deep networks effectively. *He Initialization* helps maintain a proper variance of activations, which keeps gradients at a manageable scale. Bayesian optimization was employed for hyper-parameter tuning for a few models to obtain higher accuracy. The justification for the selection of the classification models is described as follows:

- MobileNet-v2 based Classification: MobileNetV2 is designed to be computationally efficient, making it well-suited for resource-constrained environments such as mobile devices or edge computing devices. This efficiency allows it to process raw audio data with reduced computational overhead, enabling real-time or near-real-time applications, as it utilizes depth-wise separable convolutions consisting of two layers. The layers of the MobileNet architecture can be modified to extract features from raw audio signals. Its depth-wise separable convolutions are effective in capturing relevant patterns and characteristics present in the audio waveform, enabling accurate classification or analysis of audio data.
- YAMNet-based Classification: YAMNet model can process raw audio data directly, enabling efficient feature extraction and classification. This architecture comprises several layers of 1D convolutions, batch normalization layers, and activation functions, culminating in a linear classifier for feature extraction and classification. Notably, the YAMNet model employs depth-wise separable convolutions, which enhance computational efficiency while preserving representational capacity.
- RawNet-based Classification: RawNet model incorporates various components, including residual blocks and convolutional layers, to effectively process raw audio data and extract meaningful features for classification. The Residual blocks enable the model to learn residual mappings, which can help prevent the gradients from diminishing as they propagate through the network. Each residual block consists of two convolutional layers with batch normalization and LeakyReLU activation functions. These blocks facilitate the extraction of hierarchical features from the input audio data while preserving important information.

The direct use of raw audio features captures the intricate temporal and spectral details present in elephant vocalizations. However, the inclusion of additional features, such as energy, entropy, spectral bandwidth, zero-crossing rate (ZCR), and fast Fourier transform (FFT)-based descriptors, can certainly enhance the analysis and offer complementary insights. In this study, we prioritized raw waveform analysis to eliminate potential biases introduced by feature extraction processes and to fully leverage the power of deep learning architectures. While feature extraction methods like wavelet transform or quantile transform are powerful for emphasizing specific signal attributes, they often introduce biases or fail to capture the full complexity of bioacoustic signals, particularly when applied to variable and context-rich elephant vocalizations. Raw audio inputs allow models to learn discriminative representations directly from the data, often resulting in better generalization to unseen contexts.

The second approach, audio spectrogram-based processing leverages feature extraction methods, including Mel-Frequency Cepstral Coefficients (MFCC) and Chroma Constant-Q Transform (CQT), and models such as MobileNetV2, YAMNet, SVM, and ResNet18. By extracting these relevant features, our dataset was meticulously prepared for subsequent model development and evaluation. We conducted a detailed performance evaluation for caller-type classification for both approaches, followed by a comparative analysis to determine the efficacy of raw audio processing versus audio spectrogram processing.

### 3.4. Proposed ElephantCallerNet for Raw Audio Classification

**Figure 3:**
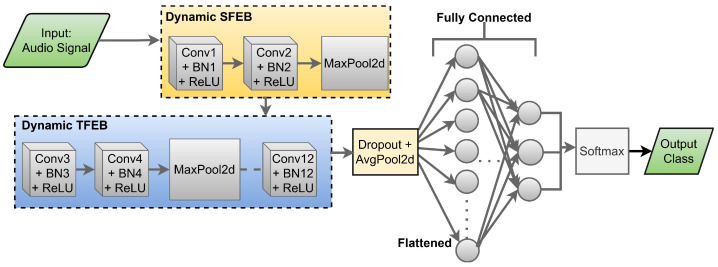
Architecture of ElephantCallerNet Model.

The ACDNet theory, or Acoustic Complexity Descriptor (ACD), is a method used for analyzing the acoustic complexity of envoronment sound signals [9]. The proposed ElephantCallerNet model, inspired by the ACDNet architecture, incorporates similar design principles and techniques to facilitate elephant call-type classification. Figure 3 illustrates a design of the ElephantCllerNet model. Initially, the model extracts static and dynamic features from raw audio data, utilizing spatial feature extraction block (SFEB) and temporal feature extraction block (TFEB), respectively.

First, the SFEB focuses on extracting spatial features from individual frames. They are designed to enhance the model’s ability to recognize patterns within a spatial context. It utilizes convolutional operations together with pooling layers to reduce dimensionality while retaining essential spatial information. Also, it incorporates dropout regularization to prevent overfitting. It is important to use SFEB before TFEB, as SFEB extracts fine-grained frequency details, that can be merged during TFEB otherwise, which leads to performance degradation. Thus, this strategy discourages the network from becoming overly reliant on certain features, promoting the learning of more resilient representations.

Then, the TFEB is designed to capture temporal dependencies in sound data such as variations over time and rhythmic patterns. It utilizes attention mechanisms to focus on relevant parts of the input sequence, facilitating the extraction of meaningful features over time. TFEB is implemented as a sequence of convolutional layers, batch normalization, and ReLU activation functions within the ElephantCallerNet architecture. This module uses both MaxPooling and AveragePooling layers to downsample the feature maps, allowing the network to focus on the important information while reducing computational complexity and over-fitting.

Since SFEB and TFEB produce feature tensors with different dimensions, the features are permuted to ensure compatibility by rearranging the dimensions. Once the feature tensors are aligned, they are concatenated along the channel dimension. After concatenation, the combined feature tensor contains all the extracted features. The feature integration and classification process is handled by incorporating convolutional layers along with batch normalization and ReLU activation functions. Max pooling operations are applied to aggregate spatial information across feature maps, while an optional dropout layer helps prevent over-fitting during training randomly removing units from the neural network. At the final stage, the feature representation obtained from the convolutional layers undergoes processing via a fully connected layer, that performs linear transformations to align the features with the respective output classes. Subsequently, the output layer utilizes a softmax activation function for the classification of different types of elephant calls.

We have implemented the models using PyTorch 2.3.0+cu121, with some auxiliary Keras libraries utilized in the process. All models were developed within the PyTorch framework. The experimentation and training were conducted on a Lambda Cloud GPU 1× A6000 server, with the CUDA Version 12.2 environment. The server hardware specifications include an Intel(R) Xeon(R) Platinum 8480+ processor.

### 3.5. Web Application Development

**Figure 4:**
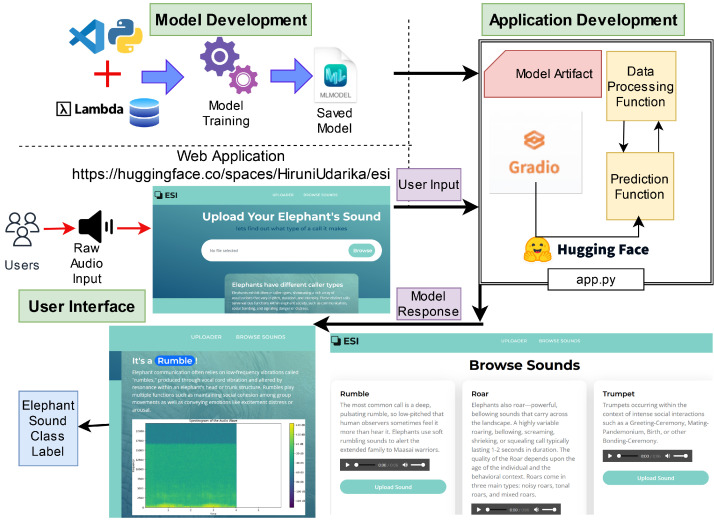
Elephant Monitoring System Web Application.

We developed a web application based on the proposed ElephantCallerNet model and deployed in https://huggingface.co/spaces/HiruniUdarika/esi (accessed on 1 December 2024). As shown in Figure 4, we introduced a portal hosted on a Lambda server, enabling users to upload audio files for caller-type classification. This implementation is a pivotal step towards the real-time application of our research findings.

In Figure 4, the edge device is the component that runs the trained model, minimizing computational demands on the device. A cloud server is a component that facilitates communication between the edge device and the database, enabling model updates and facilitating data storage and processing. User Interface (UI) serves as the primary point of interaction for users, providing a platform to upload and analyze audio recordings of elephant calls. The flow of data and operations is depicted as follows:

- Raw audio input: Users submit audio recordings of elephant calls through the UI.
- Prediction: The edge device, equipped with the trained model, analyzes the uploaded audio, generates predictions, and transmits them to the cloud server.
- Response: The cloud server integrates with a database to provide additional information and context for the predicted call types. This information is then relayed back to the user through the UI.

This architecture demonstrates the system’s ability to perform real-time elephant call classification on edge devices, offering a user-friendly interface and leveraging the power of cloud computing for enhanced capabilities. This is a simple example Application demonstration that needs to be enhanced further.

## 4. Results

This section presents the results with a comparative evaluation among the models considered in this study, covering a wide range of combinations.

### 4.1. Assessment of Model Performance with Different Augmentation Levels

**Figure 5:**
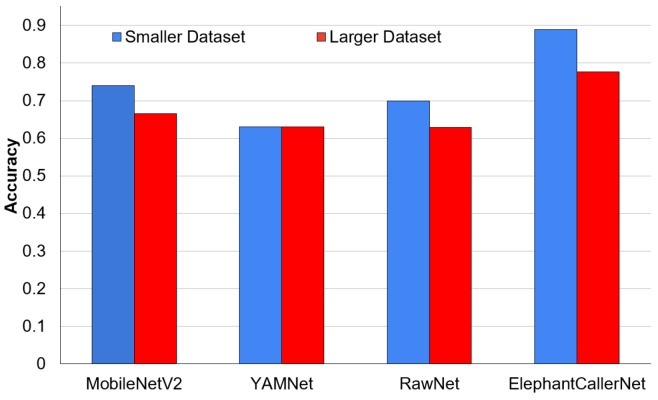
Comparative analysis of accuracy for direct raw audio processing in different augmentation levels.

Initially, we assessed the accuracy of different models in direct raw audio processing, to identify the use of the augmentation levels. Figure 5 shows the comparative performance of models when trained on a larger, highly augmented dataset (1082 samples) versus a smaller, minimally augmented dataset (317 samples). This analysis aims to understand the impact of the size and diversity of the training data for the accuracy and robustness of elephant caller-type classification. It can be observed that the smaller dataset with fewer augmentations performed reasonably well, the larger dataset with extensive augmentations using time stretching and pitch shifting have not yielded a significant improvement in accuracy. This suggests that the proposed ElephantCallerNet model performs well with less data, addressing the scarcity of elephant caller-type data. The analysis reveals that while data augmentation is a useful technique, it must be applied wisely to avoid degrading the quality of the dataset. The presence of artifacts from excessive augmentation can lead to lower performance metrics.

Similarly, we assessed the performance of the second pipeline, that utilized feature extraction followed by classification. As shown in Table 4, the larger dataset with high augmentation levels has shown averagely improved performance compared to the smaller dataset with less augmentation. However, ResNet18 has shown the highest accuracy of 78% for the smaller dataset. Here, YAMNet has the shortest inference time, making it the most efficient model in speed, while SVM has the longest inference time.

Accordingly, it can be observed that dataset augmentation has not significantly improved the performance for raw audio-based classification. As a result, the evaluation continued with smaller datasets to demonstrate the model’s effectiveness with limited data with the proposed ElephantCallerNet model. Nonetheless, the model also performed well on larger datasets. For comparison, the results from the smaller dataset were used for spectrogram-based/audio-visual classification as well.

### 4.2. Results Analysis of Direct Raw Audio Classification Using a Smaller Dataset

#### 4.2.1. Comparison of Configuration Setting with Performance in Direct Raw Audio Classification

The proposed solution in this study addresses multi-classification, considering commonly used three distinct elephant call types, namely roar, rumble, and trumpet, increasing experimental complexity, unlike the related studies, which often focused on binary or single-caller classification.

Table 5 outlines experimental setups and hyper-parameter configurations, ranking models by test accuracy, learning rate, and parameter count. The classification accuracy of roar caller-type is lower for the existing models, where the proposed model classified it accurately. Thus, extensive analysis revealed that the ElephantCallerNet model outperformed other models in terms of accuracy in distinguishing elephant calls for smaller datasets.

Additionally, MobileNetV2 has the shortest inference time at 0.20 s, making it ideal for real-time applications. ElephantCallerNet follows with an inference time of 0.76 s, providing a good balance between speed and performance for applications where moderate latency is acceptable. In contrast, YAMNet (1.76 s) and RawNet (1.72 s) exhibit significantly longer inference times, limiting their usability in time-sensitive contexts. Models with more parameters typically have longer inference times, affecting their usability in real-time applications. Thus, the choice of model should align with the application requirements.

When deploying such an application as an embedded system, it is vital to analyze the computational cost and complexity of the models. Here, FLOPS (Floating Point Operations Per Second) or GFLOPS (Giga Floating Point Operations Per Second) measures the number of mathematical calculations a model can perform in a second, especially in deep learning contexts. It includes operations like addition, subtraction, multiplication, and division involving numbers with decimals. FLOPS helps evaluate the computational performance of hardware or models and determines whether a model is suitable for specific hardware, such as mobile devices or edge computing platforms.

We showed the results for two scenarios, a single audio input and a batch of 32 audio inputs, reflecting both individual and batch processing efficiency. YAMNet and RawNet prioritize richer feature extraction capabilities, resulting in higher GFLOPs, which may be better suited for high-performance computing systems. MobileNetV2 and ElephantCallerNet, being lightweight, are optimized for real-time or edge applications where computational resources are limited. Although MobileNet V2 has the lowest GFLOPS requirement, making it ideal for resource-constrained environments such as mobile devices or real-time systems, accuracy is often the primary criterion for model selection, particularly in tasks like elephant sound classification, where precision is critical for real-world applications such as conservation and monitoring. ElephantCallerNet, with its comparatively low GFLOPS requirement and high accuracy, strikes an excellent balance between computational efficiency and performance, making it the most suitable choice for this task.

#### 4.2.2. Individual Model Performance in Direct Raw Audio Classification

**Figure 6:**
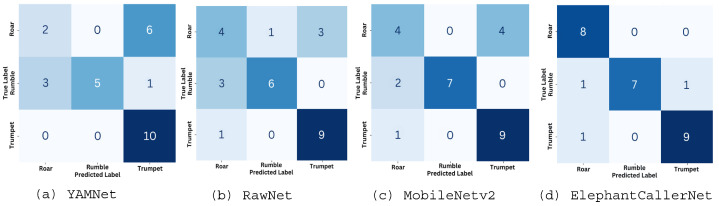
Confusion Matrix for Direct Raw Audio Processing using Small Dataset.

**Figure 7:**
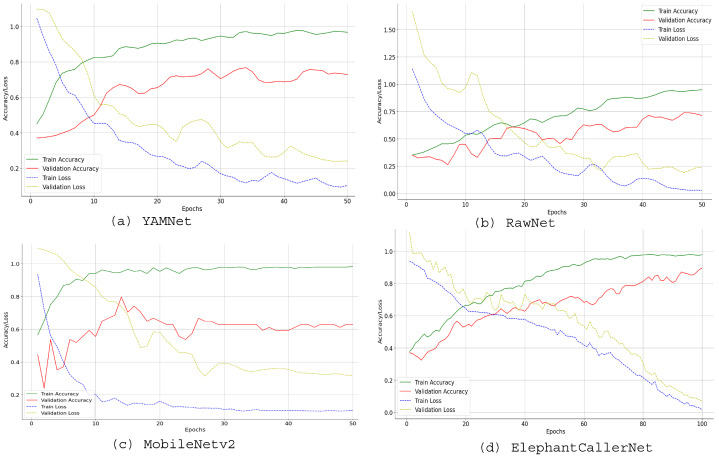
Learning Curves for Direct Raw Audio Processing using Small Dataset.

Figure 6 and Figure 7 show the values of the confusion matrix and the learning curves, respectively, for direct raw audio processing using small datasets, utilizing the four models (a) YAMNet, (b) RawNet, (c) MobielNetv2, and (d) the proposed ElephantCallerNet. Considering Figure 6, it can be seen that, except for the proposed ElephantCallerNet, the other models have misclassified several caller types; for instance, the roar sound is predicted as trumpet caller types. Thus, compared to other models, ElephantCallerNet has classified the caller types with high accuracy.

Additionally, Figure 7 confirms the high performance of the proposed model. For example, considering the deviation between the training and validation accuracy curves, YAMNet and MobileNetv2 show more overfitting, where the model performs well on training data but poorly on unseen validation data. Although, RawNet indicates low overfitting, it does not show high prediction accuracy. In ElephantCallerNet, the training and validation accuracy start low, but trend upward, reaching approximately 0.9 by epoch 60, indicating high performance. Moreover, the close alignment of training and validation metrics suggests effective generalization to unseen data, while minimal deviation indicates low overfitting. Thus, the proposed ElephantCallerNet shows efficacy and generalization capabilities in classifying call-types from raw audio data by integrating static and temporal features, achieving better performance than baseline methods.

#### 4.2.3. Comparison of Model Performance in Direct Raw Audio Classification

**Figure 8:**
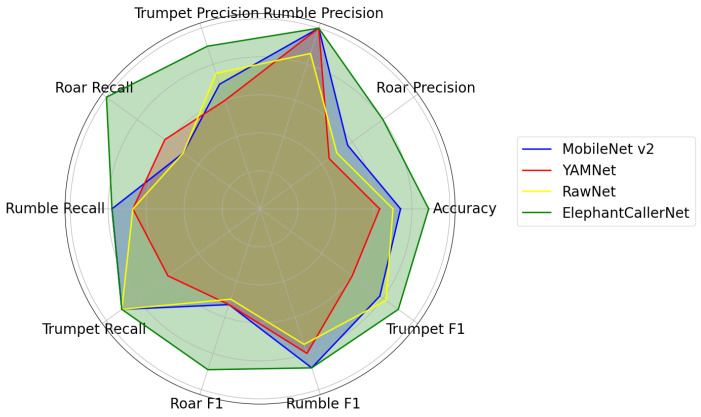
Performance Comparison for Direct Raw Audio Classification.

A radar chart effectively highlights the performance aspects of each model, underscoring the need to align model selection with specific application requirements, and balancing overall accuracy with class-specific metrics. Figure 8 presents a radar chart visualizing performance metrics (accuracy, precision, recall, and F1 score), for the four models (MobileNetV2, YAMNet, RawNet, and ElephantCallerNet), across different caller-type classes (roar, rumble, and trumpet). This visualization effectively highlights each model’s strengths and weaknesses, aiding in selecting the most suitable model for specific applications. ElephantCallerNet consistently excels, achieving high precision and near-perfect recall and F1 scores for rumble and trumpet classes, demonstrating its effectiveness in these areas.

In contrast, MobileNetV2 exhibits balanced performance across all classes, with an overall accuracy of 0.74 and strong F1 scores for rumble, indicating its reliability for applications needing stable performance across categories. YAMNet struggles with accurately classifying the roar category, as indicated by its lower precision (0.45) and F1-score (0.53). However, it excels in detecting the rumble class, suggesting potential optimization for certain sound types such as roar caller type. Moreover, RawNet does not consistently outperform other models. It achieves moderate accuracy (0.70), with precision and recall varying by class; notably, it has good precision for trumpet (0.75), but a lower recall for rumble (0.67). This suggests that RawNet requires optimization to enhance its overall reliability. Accordingly, ElephantCallerNet excelled overall, while MobileNetV2 and YAMNet performed well in specific areas.

#### 4.2.4. Comparison of Model Size, Complexity, and Accuracy in Direct Raw Audio Classification

**Figure 9:**
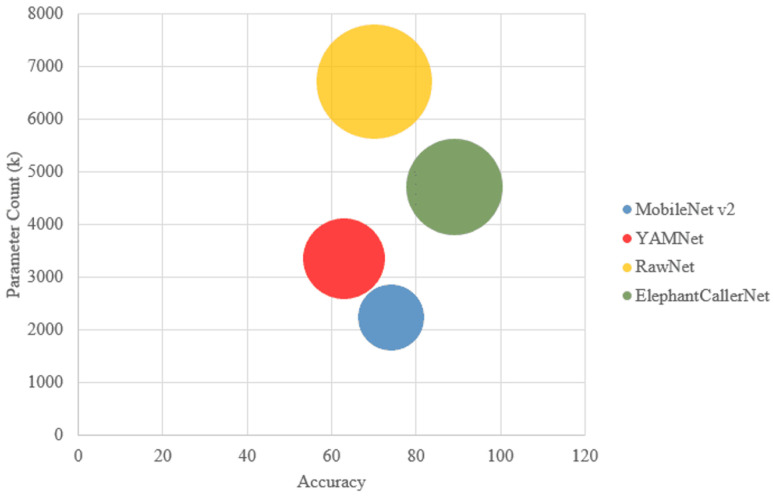
Analysis of Model Accuracy, Parameter Count, and Size across in Raw Audio Classification.

In the context of performance optimization, resource management, and model deployment, it is important to compare the model size, parameter count, and accuracy as shown in Figure 9. Model size is the overall memory acquired by the model, which is influenced by its architecture and its number of parameters. Parameter Count indicates the complexity of the model together with the capability of learning intricate patterns. Generally, larger models with more parameters require more computational resources and may achieve higher accuracy, while smaller models are more efficient but might yield lower accuracy. However, the increase in the parameter count may lead to overfitting and high consumption of computational resources and time. Accordingly, achieving high accuracy with fewer parameters can indicate a well-optimized model. Thus, a balance must be reached between model complexity and performance. Smaller models are easier to deploy and require less bandwidth for transmission when deploying in edge devices.

The proposed ElephantCallerNet showed the highest accuracy of 89%. In this comparative study, there is no clear correlation between model size and accuracy. Despite RawNet having the largest size (6.39 million parameters), its accuracy is lower than that of ElephantCallerNet, which has a smaller size (4.49 million parameters). This indicates that model size alone may not determine accuracy in this scenario. Despite having the least parameters, MobileNetv2 shows good accuracy, indicating effective optimization. In contrast, RawNet, which has the largest parameter count, does not outperform ElephantCallerNet, emphasizing that increased parameters do not guarantee better performance. Thus, efficient parameter usage and architectural design are essential, as evidenced by RawNet’s lower accuracy despite its size. These findings indicate that the ElephantCallerNet model outperforms other raw audio analysis models, emerging as a compact and lightweight alternative in terms of size and parameter count.

### 4.3. Results Analysis of Spectrogram-Based Classification Using Smaller Dataset

This section shows the results obtained by the process of feature extraction followed by the classification. We generated audio-visual representation for selected models, namely MobileNet, YAMNet, ResNet18 and SVM classifier. As mentioned in Section 4.1, although spectrogram-based models performed well with larger datasets, here we focus on smaller datasets for consistency of the comparison purpose, as the larger augmentation did not significantly improve accuracy. As mentioned in the methodology, we used MFCC and Chroma CQT features during feature extraction.

**Figure 10:**
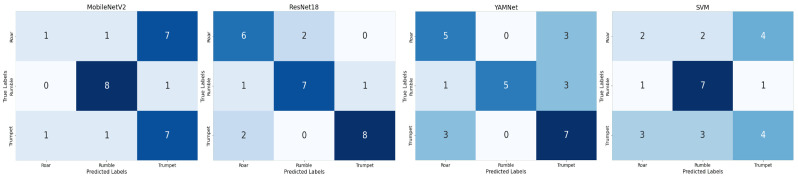
Confusion Matrix for Spectrogram Analysis using MobileNetv2, ResNet18, YAMNet, and SVM.

**Figure 11:**
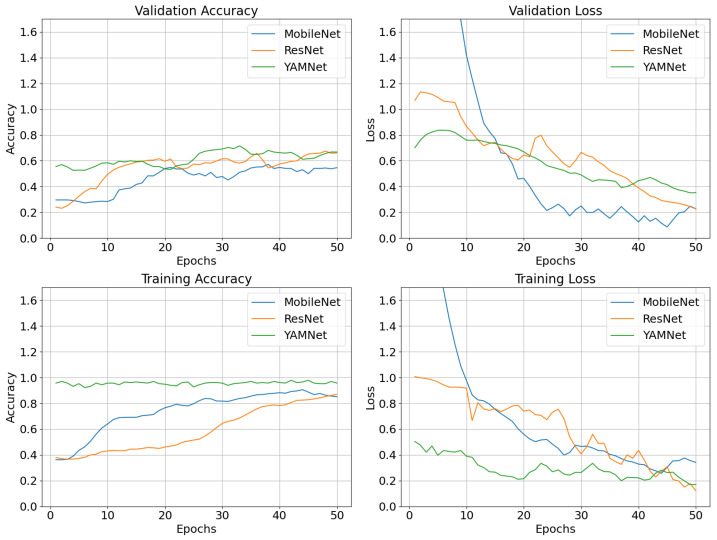
Learning curves for spectrogram-based classification using ResNet18, MobilnetV2, and YAMNet.

As shown in Figure 10, the confusion matrixes reveal that ResNet18 has shown the best performance by identifying more correct predictions compared to other models. MobileNetV2 has not performed well with the roar class, achieving only one correct prediction while classifying the rumble caller-type accurately. YAMNet also showed average performance, while SVM has generated low performance for roar and trumpet classes. Moreover, Figure 11 shows the validation accuracy, validation loss, training accuracy, and training loss for the models MobileNetv2, ResNet18, and YAMNet. All three models showed an average accuracy.

Furthermore, Table 6 states a comparison of the spectrogram-based classification with the related studies with different elephant sound datasets. The experiments conducted in this study utilized the public datasets from Asian Elephant Vocalizations Dataset (Sri Lanka) [31], ElephantVoices Dataset [32], and SoundCloud [33], considering only roar, rumble, and trumpet caller types. Most of the related studies have not explicitly specified the used datasets, and have used private datasets that contains vocalization from Sri Lankan [28], Asian, and African elephants [29], covering all the elephant sounds. It can be seen that elephant sound classification accuracy varies based on the considered dataset and the used techniques, and YAMNet and SVM showed better results.

### 4.4. Raw Audio vs. Spectrogram-Based Classification

In order to compare the performance of raw audio processing methods with state-of-the-art spectrogram-based approaches and feature-extraction models combined with machine learning classifiers, additional experiments were conducted. The comparative study presented in this study revealed the performance of different elephant sound classification techniques and augmentation levels. Direct raw audio-based classification and the spectrogram-based classification offered unique advantages and can be evaluated based on accuracy and effectiveness. For the spectrogram representation followed by the classification approach, we extracted Chroma CQT and MFCC features and trained models using MobileNetV2, SVM, ResNet18, and YAMNet. The characteristics of the audio signals and the selection of the model architecture is important to obtain the optimal solutions.

**Figure 12:**
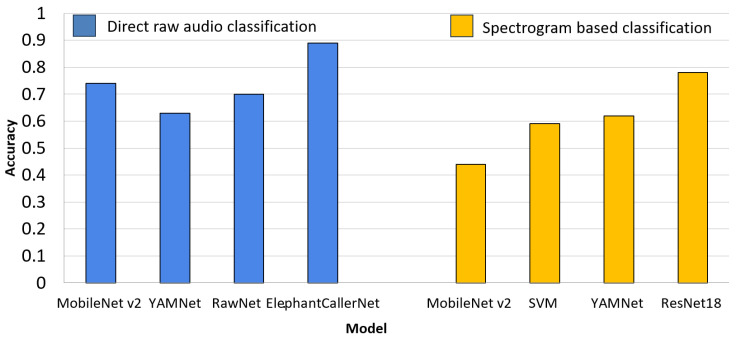
Comparison of Direct Raw Audio Classification vs. Spectrogram-based Classification.

Figure 12 illustrates accuracy comparisons of the model considered in the two approaches. The proposed ElephantCallerNet achieved the highest accuracy of 89%, with a model size of 4.49 MB and an inference time of 0.76 s. Notably, the inference time for audio-visual processing is significantly higher than for raw audio processing due to the additional feature extraction step, where raw audio processing feeds the waveform directly into the neural network, allowing the model to learn relevant features during training. Additionally, our results suggest that while accuracy varies across models and feature combinations, raw audio processing with models like MobileNet V2 and RestNet18 also showed good results.

Moreover, considering computational complexity and performance as given in Table 5, the proposed ElephantCallerNet model shows the suitability for edge deployment with constrained settings. Further, Table 7, states the comparison of the performance of various models on the same datasets. The performance of raw audio-based methods, such as ElephantCallerNet, is significantly better than spectrogram-based methods like MobileNetv2 and ResNet18, which achieved accuracies of 44% and 78%, respectively, but required larger model sizes and longer inference times. Traditional classifiers like SVM, using MFCCs and chroma features, demonstrated lower performance with an accuracy of 59%, a model size of 47 MB, and an inference time of 8.76 s. It can be concluded that the models trained on raw audio can directly learn from temporal and spectral patterns in waveforms, leading to superior performance and reduced computational overhead. While spectrogram-based methods and traditional classifiers remain viable for some applications, their reliance on preprocessing and higher resource requirements make them less suitable for real-time or resource-constrained environments.

## 5. Discussion

### 5.1. Study Contributions

In our study, we evaluated various audio-processing architectures to determine their effectiveness in recognizing elephant calls. A novel architecture, ElephantCallerNet is proposed to classify elephant sounds directly without converting into a spectrogram-based audio-visual representation, making it suitable to deploy in resource-constraint edge devices with reduced model size and inference time. Additionally, we evaluated the results obtained from the same dataset with different levels of augmentations, and found that the models produce high results for certain augmentation levels including time stretching and pitch shifting as shown in Figure 5. Subsequently, we have shown that the models perform well with small datasets, supporting data scarcity. Importantly, we performed a comparative study along two pipelines. The direct raw audio classification is compared with MobileNetv2, YAMNet, and RawNet, which are lightweight architectures that are suitable for edge deployment. The spectrogram-based (MFCC, Chroma_cqt) classification is assessed using MobileNetv2, YAMNet, ResNet18, and SVM classifiers, which support efficient feature extraction. While most literature focuses on single caller types or binary classification, our models classify three distinct types namely roar, rumble, and trumpet. Table 5 summarizes the experimental configurations and accuracies, demonstrating that our proposed model excels in raw audio analysis. Notably, ElephantCallerNet is compact, making it a lightweight alternative without sacrificing performance.

The ElephantCallerNet model is designed for elephant call classification, with features such as custom pooling for flexible dimensionality reduction, dropout regularization to address overfitting in smaller dataset, and optimized feature extraction. Its parameterized TFEB module composition allows flexibility based on input characteristics, enhancing the capture of elephant call nuances. The convolutional and batch normalization layers improved its capability to learn complex audio features. Additionally, it utilized Kaiming Normal initialization for ReLU activations for fast convergence, optimizing weights for faster convergence. The associated quantization techniques helped to reduce the model size and increase the inference speed on edge devices. In output processing, the model applied softmax to output logits, ensuring a meaningful probability distribution for more reliable classification. These innovations collectively enhance the performance of the proposed model in identifying elephant calls, making it a robust and generalized model. The proposed ElephantCallerNet achieved a balance between performance and computational efficiency, attaining an accuracy of 89% with a compact model size of 4.49 MB and 4.7 million parameters. Additionally, a user-friendly web-based application was developed to classify elephant caller types from uploaded audio files, facilitating real-time monitoring and conservation efforts.

### 5.2. Comparison with Related Studies

Most studies in the literature primarily address binary classification, distinguishing between elephant vocalizations and non-elephant sounds or focusing solely on a given caller-type. In contrast, our experiments classified three distinct elephant call-types namely, roar, rumble, and trumpet, adding complexity to our approach. Despite this, our results indicated that raw audio processing can match or even exceed the performance of traditional spectrogram-based classification models. This underscores the importance of raw audio in the bioacoustics domain, providing a robust method for elephant call classification. Given the scarcity of literature on elephant caller type classification, we compared our results with those on elephant vocal identification.

Although, there are no direct related studies to compare with those that have used the same datasets and caller types we have considered, we considered studies with elephant sound classification with different datasets and caller types to provide an overview of the comparison. Previous research in elephant caller-type identification has largely focused on spectrogram-based models [7,24,28,29]. This study introduced a raw audio classification model and the comparative analysis is shown in Table 7. The experiments conducted in this study utilized the public datasets from Asian Elephant Vocalizations Dataset (Sri Lanka) [31], ElephantVoices Dataset [32], and SoundCloud [33], considering only roar, rumble, and trumpet caller types. Most of the related studies with spectrogram-based classification have not explicitly specified the used datasets, and have used private datasets that contain vocalization from Sri Lankan [28], Asian, and African elephants [29], covering all the elephant sounds or only rumble caller type. It can be seen that elephant sound classification accuracy varies based on the considered dataset and the used techniques, and overall, YAMNet and SVM showed better results.

Our spectrogram-trained models have lower success rates compared to similar studies such as YAMNet [28] that achieved the highest accuracy of 98%. Since, audio spectrogram processing transforms raw signals into visual representations, such as MFCCs and Chroma CQT, which help the model identify patterns, it may result in high accuracy levels. However, both processing approaches have merits. Raw audio processing, as evidenced by ElephantCallerNet’s high accuracy, demonstrates that models can effectively learn from raw waveforms. Meanwhile, spectrogram processing can achieve high accuracy with advanced models and feature extraction. However, it showed high model sizes. For example, The SVM classifier consumed high model size due to the usage of the non-linear kernel, which gives higher accuracy with complex spectrogram patterns compared to the linear kernel. The choice between these methods depends on specific application requirements, computational resources, and the desired balance between simplicity and performance. It can be concluded that the raw audio models are notably lighter than other models, highlighting the potential of raw audio processing to achieve comparable accuracy with fewer trainable parameters. Thus, based on our findings, it can be concluded that the proposed ElephantCallerNet based direct raw audio classification achieved high performance compared to spectrogram-based methods, and can be deployed in edge devices with constrained resources.

### 5.3. Application of Research Findings

The proposed elephant caller type classification model can be facilitated in several directions. This can be deployed in real environments supporting wildlife conservation and management, particularly for preserving elephant populations and their habitats. The accurate classification of elephant vocalizations into distinct caller types such as rumble, roar, and trumpet offers insights into their behavior and communication. This information enables conservation practitioners to monitor populations, track individuals, and assess group health. Understanding vocalizations also helps address threats like poaching and habitat loss. Additionally, this solution can be used to monitor elephant activity within their habitats, providing valuable data for habitat management. Acoustic monitoring identifies areas of high activity and migration routes, guiding land-use planning and evaluating conservation interventions.

Moreover, real-time classification of vocalizations supports early warning systems for human–elephant conflict mitigation, allowing stakeholders to anticipate and prevent potential issues. Identifying caller types linked to aggression or distress helps implement targeted conflict reduction measures. Such systems could also be implemented in Zoos to enhance visitor understanding of animal behaviors, enriching educational experiences. Additionally, insights gained from vocalization patterns may offer pre-alarms for natural disasters, highlighting their broader applicability. In summary, our methodology enhances understanding of elephant behavior and informs conservation decisions, promoting coexistence between elephants and humans in shared landscapes.

### 5.4. Study Limitations and Challenges

This research on elephant caller-type classification presented notable advancements, yet several limitations and avenues for future exploration merit attention. Firstly, the datasets employed may not fully capture the variability in elephant calls, which could hinder the model’s generalizability. While the initial dataset considered for this study comprises 235 samples, we utilized advanced data augmentation techniques such as pitch shifting, time stretching, and background noise addition. These methods effectively expanded the dataset and introduced variability to improve the model’s robustness. Studies have shown that carefully crafted data augmentation can mitigate the challenges of limited datasets, particularly for domain-specific tasks like wildlife sound classification [34]. Elephant vocalizations can indeed vary based on individual characteristics such as age, sex, and emotional state [35]. Our current dataset focuses on classifying three primary call types (rumbles, trumpets, and roars) and does not yet account for these demographic variations. Our collected dataset predominantly includes audio recordings of adult male and female elephants, as they are the most observed and documented demographic groups in field studies. While these recordings provide valuable insights into general vocalization patterns, they do not encompass the full diversity of elephant vocalizations across age groups. Additionally, we adopted lightweight neural network architectures tailored to small datasets, ensuring the models avoid overfitting and generalize well. However, our model is designed with scalability in mind, allowing for retraining with additional labels for age and sex distinctions when such labeled data becomes available. Additionally, our lightweight model architecture, ElephantCallerNet, is designed to be modular and can be retrained on datasets with enriched metadata, making them suitable for deployment in resource-constrained environments.

Moreover, our comparison with literature data is limited due to the lack of specific studies on three caller-type classifications, relying instead on general elephant sound identification research. The implementation of models like ElephantCallerNet offers room for optimization tailored to the audio characteristics of elephant calls. Additionally, while promising results were achieved, the model’s generalization across diverse environmental conditions and elephant populations has not been explored and warrants future assessment. Exploring alternative feature extraction techniques could enhance classification performance, while addressing noise robustness is crucial for accurate identification amid environmental interference. Moreover, incorporating the knowledge of bioacoustics experts is essential for refining methodologies and ensuring that insights from the field are effectively integrated into future research. Together, these areas represent critical opportunities for further development in elephant vocalization analysis.

### 5.5. Future Possible Extensions

Several directions are possible with the extensions of the proposed model. Expanding the dataset to include diverse elephant caller types enhances its comprehensiveness, capturing a wider array of acoustic variations that improve the model’s generalizability. The application of Mix-up techniques will enable the model to learn effectively across sound variants, enhancing its robustness to variations in acoustic features. Moreover, optimizing the model for deployment on edge devices is crucial due to their limited computational resources [10,36]. This optimization involves techniques such as quantization, which reduces the precision of model parameters, and pruning, which removes redundant connections, maintaining performance while minimizing memory usage. Model compression methods, like distillation, transfer knowledge from larger models to smaller ones, preserving predictive capabilities while reducing size. Additionally, leveraging specialized hardware accelerators, or dedicated neural processing units can significantly enhance the inference speed. By optimizing the model architecture and inference pipeline for specific hardware, we can achieve greater efficiency in real-world applications. Together, these strategies not only bolster the scientific robustness of the models but also enhance their practical applicability in conservation efforts and monitoring of elephant vocalizations.

Moreover, the application deployment in real-time scenarios can be refined, wherein elephant sounds detected by sensors or audio modules prompt immediate identification amidst background noise. Upon detection, the system would swiftly generate alert messages to households or activate alarm buzzers as necessary. By transitioning from offline file uploads to real-time detection and response mechanisms, our system could significantly contribute to early warning and mitigation efforts in areas prone to human–elephant conflicts. The integration of such a real-time application aligns with the overarching goal of our research, such that to bridge the gap between cutting-edge technology and on-the-ground wildlife conservation and management practices. Through continued refinement and deployment of real-time solutions, we can further enhance the effectiveness and efficiency of elephant monitoring and conservation initiatives, ultimately fostering harmonious coexistence between human communities and elephant populations. Furthermore, in a broader perspective, the proposed method can be utilized in other audio classification contexts in forest observatory [11]. The direct analysis of raw data instead of spectral data could enable rapid, real-time optimization of complex photonic systems [37].

## 6. Conclusions

We presented a solution for elephant sound classification using deep learning techniques. The research findings on elephant caller-type classification provide a significant advancement in wildlife conservation and management efforts. This system leverages state-of-the-art technology and deep learning algorithms to address the challenges associated with human–elephant conflicts and enhance elephant conservation strategies. In this study, we evaluated several deep learning models for audio-processing to assess their effectiveness in accurately identifying and classifying elephant calls. Our analysis included established models such as MobileNet, YAMNet, and RawNet, known for their proficiency in audio classification. We introduced our novel ElephantCallerNet model, designed to leverage unique acoustic features of elephant vocalizations, achieving an overall accuracy of 89%. We assessed the suitability of different augmentation techniques and classification pipelines with several comparative evaluations to identify the optimal solution. Unlike existing models that primarily focus on binary classification or single caller types, our approach classifies three distinct types namely roar, rumble, and trumpet, introducing a higher level of complexity. Overall, our study underscores the potential of machine learning models trained on raw audio data for effective elephant call classification. These findings suggest that similar methodologies could be applied to classify vocalizations from other animal species, providing a streamlined solution for wildlife acoustic monitoring and conservation efforts. This approach enhances the scalability and robustness of bioacoustic methodologies, contributing to the broader understanding and conservation of biodiversity.

## Figures and Tables

**Table 1:** Related Studies in Elephant Sounds Classification.

Study	Dataset	Class Labels	Feature Extractor	Classifier	Performance
Geldenhuys et al. (2024) [27]	Asian Elephant vocalization dataset and African Elephant voices	5 classes: rumble, trumpet, roar, bark, squelch	MFCC and mel-spectral feature	AST 1-to-1 sequence classifier	Precision: 73% Recall: 43.5% Specificity: 98.7%
Ranasinghe et al. (2023) [28]	Asian Elephant vocalization dataset (Rumble recordings) + AudioSet	Elephant sound or not	STFT spectrograms	Transfer learning with YAMNet	Accuracy: 97.7%
Silva et al. (2023) [29]	Asian Elephant vocalization dataset (Rumble recordings)	Elephant rumble or not	Wavelet signal decomposition, chroma_cqt, chroma_cens, melspectrogram, MFCC, spectral contrast	SVM	Precision: 84% Recall: 84%
Leonid et al. (2022) [7]	ElephantVoices.org, AnimalSounds.org, and other public internet resources	Elephant sound or not	MFCC, LPC, SSC	Parallel CNN	Accuracy: 96.2%, Precision: 89.2%
Bjorck et al. (2019) [25]	African Elephant sounds (Private)	Elephant sound or not	MFCC	CNN-LSTM	Precision: 90.8%, Recall: 96.4%
Zeppelzauer and Stoeger (2015) [24]	South Africa Elephant Sounds (Private)	Identify rumbles	Greenwood cepstrum features	SVM	Recall: 88.2%, False discovery rate: 86.3%
Clemins, Johnson et al. (2005) [4]	African Elephant sounds (Private)	5-classes (croak, rumble, rev, snort, trumpet)	12 MFCC coefficients and log energy	HMM	Accuracy: 79.7%

**Table 2:** Train and Evaluation Data Split Before Data Augmentation.

Caller Type	Raw Audio Count After Pre-Processing	Split Data (80:10:10)		
		Train	Validation	Test
Roar	77	61	8	8
Rumble	95	76	10	9
Trumpet	63	50	7	6

**Table 3:** Data Split for Training and Evaluation After Augmentation.

Caller Type	Approach 1				Approach 2			
	Total	Train	Validation	Test	Total	Train	Validation	Test
Roar	104	80	16	8	378	305	65	8
Rumble	109	80	20	9	419	370	40	9
Trumpet	104	80	14	10	285	230	45	10
Total dataset	317	240	50	27	1082	905	150	27

**Table 4:** Comparative Analysis of Performance with Audio-Visual Training for Different Augmentation Levels.

Model	Inference Time (s) (Smaller Dataset)	Accuracy (Smaller Dataset)	Accuracy (Larger Dataset)
MobileNet V2	5.85	0.44	0.55
YAMNet	3.31	0.62	0.55
ResNet18	6.40	0.78	0.70
SVM	8.76	0.59	0.70

**Table 5:** Model Configuration and Performance for Direct Raw Audio-based Classification.

Metric	YAMNet	RawNet	MobileNet v2	Elephant CallerNet
Overall Accuracy	0.63	0.70	0.74	0.89
Accuracy (Roar)	0.25	0.5	0.5	1.0
Accuracy (Rumble)	0.55	0.66	0.77	0.77
Accuracy (Trumpet)	1.0	0.9	0.9	0.9
Learning Rate	0.0002	0.00590	0.000552	0.0005
Training Batch Size	32	32	32	32
Validation Batch Size	8	8	8	8
Number of Parameters	3,351,427	6,703,619	2,227,139	4,708,682
Model Size in MB	3.2	6.39	2.12	4.49
Inference Time (seconds)	1.76	1.72	0.20	0.76
GFLOPS per audio	38.13	16.84	1.79	2.35
GFLOPS per 32 batch audios	1220.40	538.96	57.57	75.21

**Table 6:** Comparative Analysis of Model Accuracy with Audio-Visual Training.

Model	Extracted Features	Accuracy %
This Study		
MobileNet v2	MFCC, Chroma_cqt	44
YAMNet	MFCC, Chroma_cqt	62
ResNet18	MFCC, Chroma_cqt	78
SVM	MFCC, Chroma_cqt	59
From Related Literature Review		
YAMNet [28]	Mel spectrogram	97.7
SVM [29]	Wavelet-based signal reconstruction, Quantile Transformer, chroma_cqt and chroma_cens, Mel spectrogram and MFCC, spectral_contrast	82
Linear SVM [24]	Short-time spectral features	85.7
Parallel CNN [7]	MFCC, LPC, SSC, MBE	91.1

**Table 7:** Comparison of the Proposed Study Results with Related Studies.

Model	Dataset	Feature Extraction	Model Size (MB)	Inference Time (s)	Accuracy %
This Study					
MobileNetv2	Roar, rumble, and trumpet	Raw Audio	2.12	0.20	74
YAMNet	Asian Elephant Vocalizations [31]		3.2	1.76	63
RawNet	ElephantVoices Dataset [32]		6.39	1.72	70
Elephant CallerNet	SoundCloud [33]		4.49	0.76	89
MobileNetv2	Roar, rumble, and trumpet	MFCC, Chroma_cqt	8.67	5.85	44
YAMNet	Asian Elephant Vocalizations [31]		12.83	3.31	62
RestNet18	ElephantVoices Dataset [32]		42.62	6.40	78
SVM	SoundCloud [33]		47.00	8.76	59
From Related Studies					
SVM [29] (2023)	Private dataset (Asian, African)-Rumbles Class	Wavelet transf., MFCC, spectral_contrast, quantile transf., chroma_cqt, chroma_cens, Mel spectrogram,	Not Mentioned	Not Mentioned	82
Linear SVM [24] (2015)	Private dataset (South Africa)-Rumble Class	Short-time spectral features	Not Mentioned	Not Mentioned	85.7
Parallel CNN [7] (2022)	Elephantvoices.Org, AnimalSounds.Org, Soundbible.Com	MFCC, LPC, SSC, MBE	Not Mentioned	11.89	91.1
YAMNet [28] (2023)	Private Dataset (Sri Lanka)	Mel spectrogram	Not Mentioned	Not Mentioned	97.7

## Data Availability

Asian Elephant Vocalizations Dataset https://catalog.ldc.upenn.edu/LDC2010S05 (accessed on 6 March 2024). ElephantVoices Dataset https://www.elephantvoices.org (accessed on 6 March 2024). SoundCloud https://www.elephantvoices.org (accessed on 6 March 2024). GitHub Repository: https://github.com/HiruDewmi/Audio_Classification_for_Elephant_Voice (accessed on 10 May 2024).
